# 7.H. Workshop: Lessons Learned Workshop from JAHEE- the importance of evaluation

**DOI:** 10.1093/eurpub/ckac129.432

**Published:** 2022-10-25

**Authors:** Organised by: JAHEE, Chair persons: Raffaella Bucciardini (Italy), Bernadette Kumar (EUPHA-MIG)

## Abstract

Joint Action Health Equity (JAHEE) was a Joint Action financed by the Third Health Programme 2014-2020 of the European Union. It was developed in the period June 1, 2018 - November 30, 2021, and involved 24 countries. It represented an important opportunity for member states to work together to address health inequalities and achieve greater equity in health outcomes across all groups in society in all participating countries and in Europe at large. The results of JAHEE show that almost all participating countries have increased their level of action to address health inequalities and strengthened their capacities in some important policy domains. 76 actions to contrast health inequalities were started during the Joint Action and a final Consensus Policy Document was produced including a set of cross-cutting recommendations. The proposed workshop will discuss the 3-year JAHEE project in the European region and through presentations and active participation review JAHEE actions, results of internal and external evaluations and discuss the main lessons learned. The session will go beyond sharing information and disseminating results and focus on what the JAHEE evaluation has taught us and how it can help improve health equity interventions in the future.

We plan to have:

a) Presentations

1. Short introduction to JAHEE and Actions

2. Presentation Internal Evaluation

3. Presentation External Evaluation

After the presentations there will be opportunity for interactive sessions with the participants to further explore the case studies and issues raised around evaluation and equity

b) Interactive Group Discussion

• What do the JAHEE evaluations findings add?

• What aspects of the evaluation were missing? What could have been improved?

• How can the lessons learned from the evaluation be applied to interventions in equity/ migrant health?

3) Feedback from Groups

4) Summary and Conclusions

**Key messages:**

• No data no progress.

• Apply an equity lens: Evaluation in health equity actions is important for the sustainability of actions and contributes to the implementation of good practices.

